# Mitochondrial genome of a typical cavefish of *Sinocyclocheilus anatirostris*

**DOI:** 10.1080/23802359.2023.2215351

**Published:** 2023-05-20

**Authors:** Xinrui Zhao, Changting Lan, Jing Yu, Jiajun Zhou, Xueli Lu, Ning Xiao, Huaiqing Deng, Jiang Zhou

**Affiliations:** a School of Karst Science, Guizhou Normal University, Guiyang, China; b School of Life Sciences, Guizhou Normal University, Guiyang, China; c Zhejiang Forest Resource Monitoring Center, Hangzhou, China; d Guiyang Healthcare Vocational University, Guiyang, China

**Keywords:** Mitochondrial genome, *Sinocyclocheilus anatirostris*, karst, phylogenetic

## Abstract

*Sinocyclocheilus anatirostris* Lin and Luo, 1986 is a member of the endemic Chinese genus *Sinocyclocheilus* Fang, 1936, living in dark caves with absence of eyes and scales. Muscle tissue was collected from cavefish samples from Guangxi, China, and complete mitogenome was sequenced. This is the first report of the mitogenome of *S. anatirostris*. This mitogenome consists of 13 protein-coding genes (PCGs), two rRNA genes (12S rRNA and 16S rRNA), 22 tRNA genes, a control region (CR), and comprises 31.2% A, 24.4% T, 16.7% G, and 27.7% C bases. Phylogenetically, *S. anatirostris* is closely related to the *Sinocyclocheilus furcodorsalis*, and originated in the late Miocene, ∼6.07 Ma.

## Introduction

**Figure 1.**
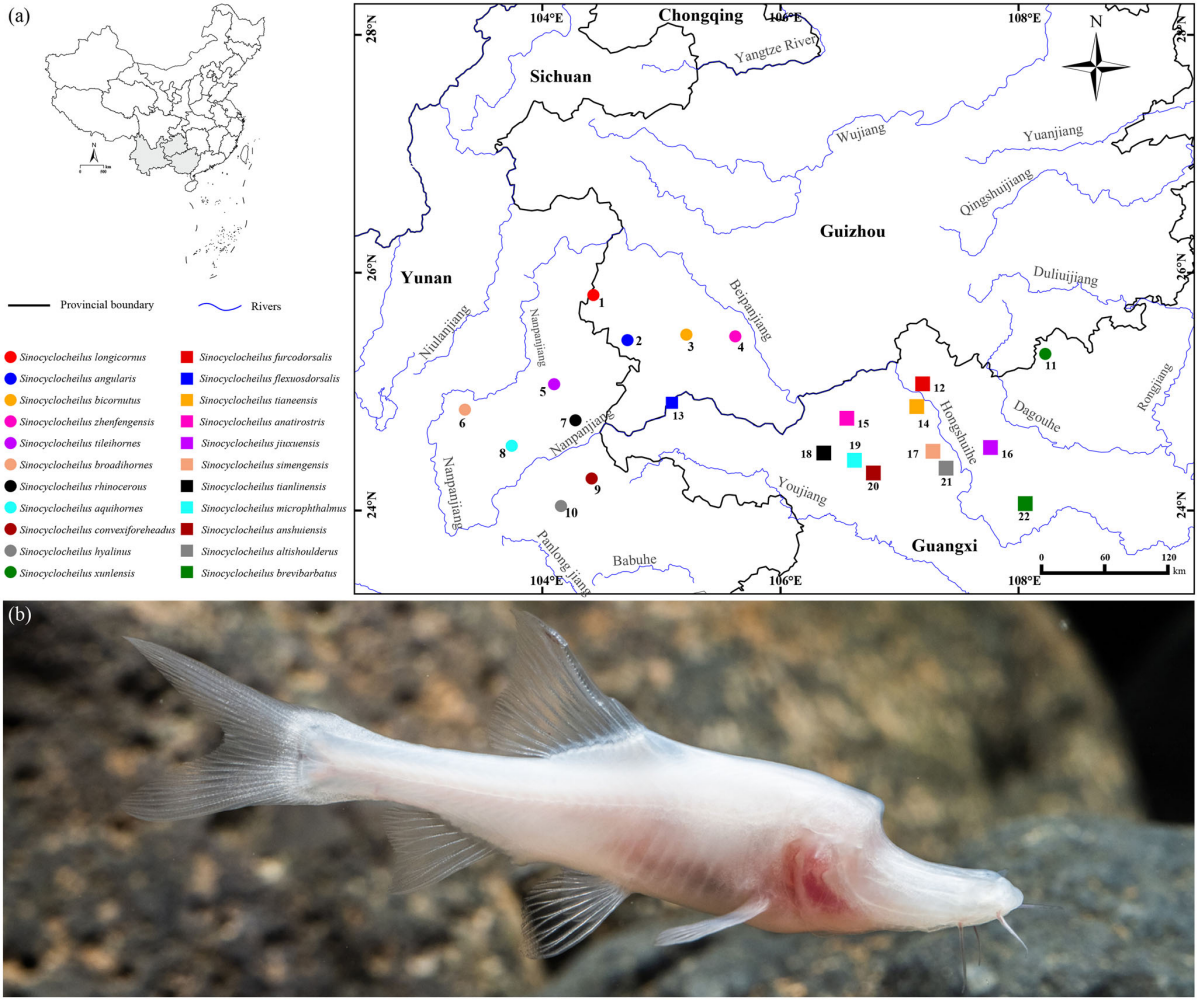
Type locality of species of the *S. angularis* and *S. microphthalmus* groups within karst areas of Southwest China (a) and ecological photograph of *S. anatirostris* (by Jiajun Zhou) (b).

The golden-line fish genus *Sinocyclocheilus* Fang 1936, endemic to the karst areas of southwest China, including Guangxi, Guizhou, Yunnan, and Hubei provinces, has 77 species recorded, 72 of which can be divided into five species groups (Fang 1936; Zhao and Zhang 2009; Wen et al. 2022; Xu et al. 2023), namely *Sinocyclocheilus angularis*, *Sinocyclocheilus cyphotergous*, *Sinocyclocheilus microphthalmus*, *Sinocyclocheilus jii*, and *Sinocyclocheilus tingi* groups. Among these five species groups, *S. angularis* and *S. microphthalmus* groups have evolved complex morphological characters adapted to the dark environment, such as horn-like structure on the back of the head (long, short, and forked), degeneration or absence of eyes and scales, and strengthening of locomotor and sensory organs (Ma et al. 2019). Currently, cavefishes of the *S. angularis* group are mainly distributed in the Nanpanjiang, Beipanjiang, and Hongshui rivers (Figure 1(a)), and the limited genetic information may limit our understanding of the evolutionary history.

*Sinocyclocheilus angularis* Lin and Luo 1986 is a typical cave species within *S. angularis* group, with a duck-billed snout, absence of eyes, scales and body pigmentation, and a short horn-like structure on the back of the head (Figure 1(b)) (Lin and Luo 1986; Zhao and Zhang 2009; Lan et al. 2013). This species has long been assessed as vulnerable by the International Union for Conservation of Nature (IUCN 2022) due to lack of sufficient recognition and protection. In this study, we sequenced the complete mitogenome of *S. angularis* and analyzed its phylogenetic relationships with other species of the congenus. The publication of this data is informative for the conservation of *S. angularis* and subsequent studies.

## Materials

Single sample tissues of *Sinocyclocheilus anatirostris* were collected in 2018 from Youping Town, Leye County, Guangxi Zhuang Autonomous Region, China (24.99766151°N, 106.48204029°E; elevation: 575 m), and kept in Guizhou Normal University, Guiyang City, Guizhou Province, China (sample ID: GZNU-YZ01, http://gznu.edu.cn, contact person: Jiang Zhou; Email: zhoujiang@ioz.ac.cn).

## Methods

Genomic DNA for sample was extracted from 95% ethanol-preserved tissue using the cetyltrimethylammonium bromide method (Allen et al. 2006). Mitogenome was sequenced at TSINGKE Biotechnology Co., Ltd. (Chengdu, China) using an Illumina Novaseq 6000 platform (Illumina, USA) with 150 bp paired-end reads. Sequencing generated 4.9 G of raw data, which were filtered using SOAPnuke 1.3 (Chen et al. 2018) to obtain 4.9 G of clean data. Clean data were *de novo* assembled using Mitoz v. 2.3 software (Supplementary Material, Figure S1). The mitochondrial genome was spliced using SPAdes 3.13 (Bankevich et al. 2012; parameters: -k 127), and the spliced sequences were blast (version: BLAST 2.2.30+; parameters: -evalue 1e-5) with the reference mitochondrial genome to identify candidate sequences for assembly. The assembled mitogenome was annotated with genes using MITOS2 (Bernt et al. 2013) and uploaded to the National Center for Biotechnology Information (NCBI, USA) under the accession number OP429108. We downloaded 30 mitogenomes from NCBI for molecular analysis, including 28 species of *Sinocyclocheilus* and two outgroups. We used MAFFT 7.471 (Katoh and Standley 2013) implemented in PhyloSuite 1.2.2 (Zhang et al. 2020) for sequence alignment and PartitionFinder 2.1.1 (Lanfear et al. 2012) to select the best-fit model (GTR + I + R). In total, 2000 ultrafast bootstrap replicates were run in IQ-tree 2.0.4 to reconstruct the phylogenetic tree. Divergence times of the two node calibrations were analyzed using BEAST 2.4.7 (Bouckaert et al. 2014) as previously described (Wen et al. 2022).

## Results

**Figure 2.**
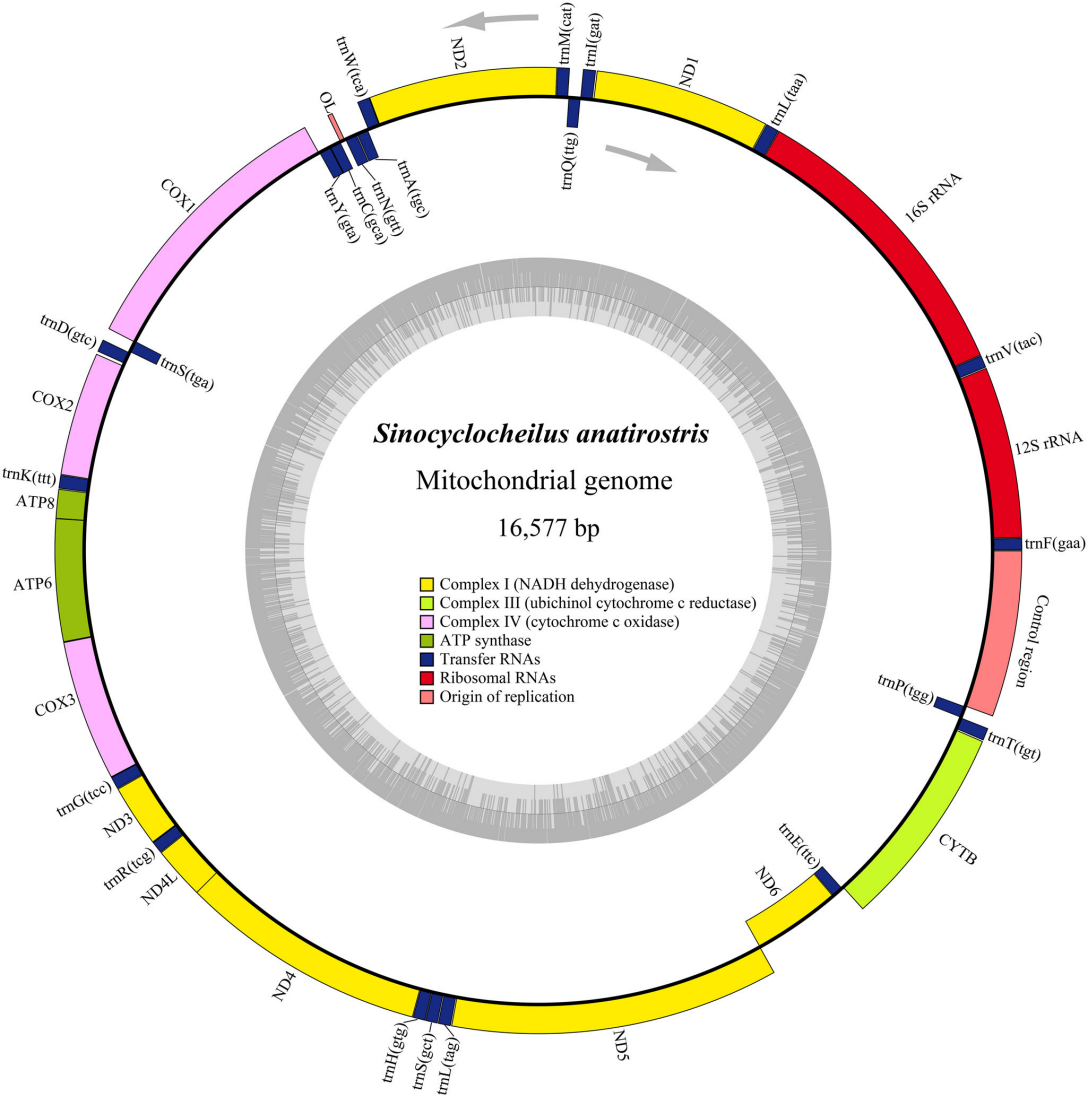
Genome map of *S. anatirostris* mitochondrial genome.

The complete mitogenome length of *S. anatirostris* was 16,577 bp (Figure 2). The mitogenome contained a control region, 13 protein-coding genes (PCGs), 22 transfer RNA (tRNA) genes, and two ribosomal RNA (rRNA) genes, which were 31.2% A, 24.4% T, 16.7% G, and 27.7% C. Among these genes, the ND6, trnQ(ttg), trnA(tgc), trnN(gtt), trnC(gca), trnY(gta), trnS(tga), trnE(ttc), and trnP(tgg) were encoded on the L-strand, while the remaining genes were encoded on the H-strand. Within the 13 PCGs, except for COI, which starts at a GTG codon, the codons of the remaining 12 PCGs started with ATG. The arrangement of these genes was similar to that of *S. angularis* (Luo and Zhang 2021). Except for ND2 and ATP8 with codon TAG, COX2, and ND4 with T– as stop codons, and COI with AGG as a stop codon, the remaining eight PCGs used TAA as a stop codon.

**Figure 3.**
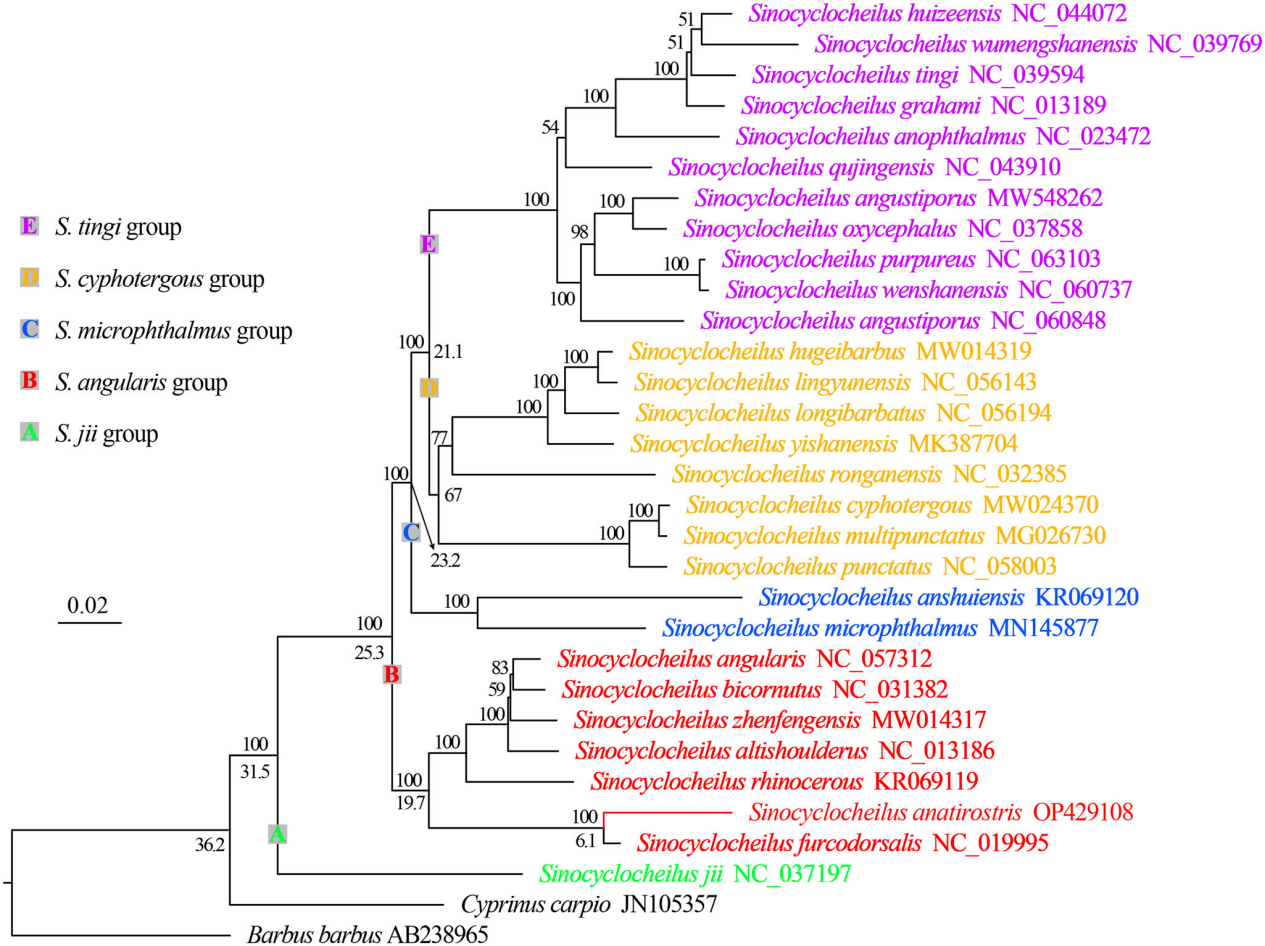
Phylogenetic tree and divergence time based on mitochondrial genome reconstruction. Above the node is the support value and below the node is the divergence time.

Phylogenetic tree reconstructed using the mitogenome shows that *Sinocyclocheilus* has six major clades corresponding to five species groups, but the monophyly of *S. cyphotergous* is not supported. Phylogenetic tree and divergence time suggests that *S. anatirostris* may be close to *Sinocyclocheilus furcodorsalis* and originated in the late Miocene, ∼6.07 Ma (Figure 3).

## Discussion and conclusions

The phylogenetic result support the division of *Sinocyclocheilus* into five species groups, i.e. *S. jii*, *S. tingi*, *S. cyphotergous*, *S. angularis*, and *S. microphthalmus* groups (Zhao and Zhang 2009; Wen et al. 2022; Xu et al. 2023), but *S. cyphotergous* group needs to be studied further using more species. Since the Miocene, the occurrence of orogeny and monsoonal climates in southwestern China have created favorable conditions for the speciation of the genus by geographic isolation (Yuan et al. 1995; Zhao and Zhang 2009; Wen et al. 2022), which has resulted in the distribution of different species of the genus in different river systems (Figure 1). *Sinocyclocheilus furcodorsalis* and *S. anatirostris* have distinct horn-like structures on the back of their head, as well as body pigmentation absence (Lan et al. 2013), while *S. angularis*, *Sinocyclocheilus bicornutus*, *Sinocyclocheilus altishoulderus*, and *Sinocyclocheilus zhenfengensis* have long or short horn-like structures, but normal or slightly degraded body coloration (Zhao and Zhang 2009; Liu et al. 2018). Based on the time of divergence, we suggest that the loss of body coloration and the emergence of the most recent common ancestor of inconspicuous or short horns occurred 19.76 Ma ago, while further development of short horns into longer horns required at least 8 Ma. Thus, we hypothesize that at least one intensification event occurred during the evolution of the horns.

We present here the first report of the complete mitogenome of *S. anatirostris*, successfully sequenced, assembled, and annotated. Phylogenetic relationships and time of origin of this species based on the mitogenome level were assessed. The mitogenome published here can be used as a basis for further studies on the phylotaxonomy, conservation biology and biogeography of *Sinocyclocheilus*.

## Supplementary Material

Supplemental Material

## Data Availability

The genome sequence data that support the findings of this study are openly available in the GenBank of NCBI at https://www.ncbi.nlm.nih.gov/ under the accession No. OP429108. The associated BioProject, SRA, and Bio-Sample numbers are: PRJNA889191, SRR21856437, and SAMN31233307, respectively.

## Vulnerable species statement

We have strictly followed the policies of the International Union for Conservation of Nature (IUCN) policies research involving species at risk of extinction, the Convention on Biological Diversity and the Convention on the Trade in Endangered Species of Wild Fauna and Flora.
